# Trajectories of peripheral white blood cells count around the menopause: a prospective cohort study

**DOI:** 10.1186/s12905-024-03344-0

**Published:** 2024-09-11

**Authors:** Qiaoyun Dai, Yaya Zhang, Xiujuan Zhao, Xueying Yang, Huayu Sun, Shouling Wu, Shuohua Chen, Jianmei Wang, Zongfu Cao, Xu Ma

**Affiliations:** 1 National Human Genetic Resources Center, National Research Institute for Family Planning, Beijing, China; 2 Graduate School of North, China University of Science and Technology, Tangshan, China; 3 Health Department of Kailuan (group), Kailuan General Hospital, Tangshan, China

**Keywords:** Menopause, Peripheral white blood cells, Chronological aging, Body mass index, Cohort study

## Abstract

**Background:**

Menopause significantly impacts the immune system. Postmenopausal women are more susceptible to infection. Nonetheless, the pattern of change in peripheral white blood cell counts around the menopause remains poorly understood.

**Methods:**

We conducted a prospective longitudinal cohort study with repeated measurements using Kailuan cohort study of 3632 Chinese women who participated in the first checkup (2006–2007) and reached their final menstrual period (FMP) by the end of the seventh checkup (2018–2020). Peripheral WBC count indicators included total white blood cells (TWBC), neutrophils (NEUT), lymphocytes (LYM), and monocytes (MON). Multivariable mixed effects regressions fitted piece-wise linear models to repeated measures of WBC count indicators as a function of time before or after the final menstrual period (FMP). Interaction and subgroup analysis were used to explore the effects of age and body mass index (BMI) on changes in WBC indicators around FMP.

**Results:**

WBC count indicators decreased before the FMP, and the reduction in TWBC, NEUT, and MON continued for 2 years following the FMP. LYM and NEUT declined during < -1 years and − 4 ∼ + 2 years relative to FMP, respectively. A reduction in MON was observed pre-FMP, extending continuously through the two-year period post-FMP. TWBC declined from − 3 to + 2 years relative to FMP, but both MON and TWBC increased during > + 2 years. The baseline age had an interaction effect on changes in WBC indicators during specific menopausal stages, except for TWBC. Individuals in different age subgroups showed distinct trajectories for NEUT, LYM and MON around the FMP. High baseline BMI had a synergistic effect on changes in specific menopause segments for TWBC, LYM, and MON. The impact of menopause on TWBC and LYM was postponed or counterbalanced in high BMI individuals. Individuals in three BMI subgroups experienced similar MON changes around FMP, and there were slight variations during < -4 years.

**Conclusions:**

Menopause was associated with count changes of peripheral WBC. The trajectories of various WBC types differ around menopause. Age and BMI affected WBC trajectory around menopause. The menopause period may represent a window of opportunity to promote immune health in middle-aged women.

**Supplementary Information:**

The online version contains supplementary material available at 10.1186/s12905-024-03344-0.

## Introduction

Menopause indicates the permanent cessation of ovarian function [1] and represents a significant milestone in ovarian aging. The menopause transition (MT) brings about a range of subtle health consequences, including an elevated risk of cardiovascular disease (CVD), diabetes mellitus, cancers, bone loss, and heightened vulnerability to infections [2, 3]. The scientific statement released by the American Heart Association in 2020 highlighted the MT as a critical window for early implementing intervention strategies aimed at reducing CVD risk [1]. Lobo et al. [4] proposed menopause presents a vital opportunity for preventive interventions, which could enhance life quality and reduce mortality. Investigating changes in risk factors and biomarkers associated with menopause is essential for understanding the onset and progression of diseases and for advancing targeted prevention and management strategies throughout a woman’s lifespan.

Menopause significantly impacts the immune system [5], leading to an increased susceptibility to infections. Post-menopausal women are more vulnerable to illnesses like HPV infection, HIV infection, and urinary tract infections, and their immune response to vaccines decreases [3, 6]. Peripheral blood immune cells play a vital role in the immune system, and different leukocyte subsets possess distinct functions. Previous small-sample studies have compared the immunophenotypes and ex vivo inflammatory response of lymphocytes or mononuclear cells in women of reproductive age versus those who are post-menopausal. These studies have revealed that menopause can alter immune phenotypes, increasing the risk of impaired immune responses in post-menopausal women [7, 8]. Given that the white blood cells (WBC) count in peripheral is a readily accessible clinical parameter, examining menopause’s impact on it serves as a valuable adjunct to our understanding of immunological changes. Some studies observed contrasting changes in postmenopausal women’s WBC counts versus premenopausal women: lowered WBC, neutrophils (NEUT), and monocytes (MON) with elevated lymphocytes (LYM) in one study [9]; trending decreases in WBC and NEUT, increases in LYM, and stable MON levels over the menopausal progression in another research [10]; and increased WBC, LYM, MON with NEUT unchanged in one research [11]. These inconsistent results, all from cross-sectional studies, necessitating prospective cohort studies to consolidate our understanding.

Age is a main confounder in menopause research. The Study of Women’s Health Across the Nation (SWAN) in the US, employing a longitudinal design, distinguishes ovarian aging from chronological aging by assessing health markers over time before and after the final menstrual period (FMP); disparate segment slopes in piecewise linear models indicate ovarian aging rather than chronological aging [12]. This design excels in examining ovarian aging effects, functioning as a robust method to study menopause effects. It has furnished the most substantial evidence linking menopause with lipid alterations and modifications in body composition [1, 12–14]. We have employed this methodology to illustrate the lipid trajectories in the Chinese population around FMP [15]. However, there is a current dearth of prospective cohort studies examining the trajectories in WBCs before and after FMP, lacking robust evidence to substantiate the impact of menopause on WBC counts.

In this study, we aimed to investigate changes in peripheral WBC count around the FMP in Chinese women based on the Kailuan cohort study. Additionally, we analyzed the effect of baseline age on WBC changes around FMP due to the overlapping effects of age and menopause. Furthermore, obesity may attenuate changes in estrogen (E2) and follicle-stimulating hormone (FSH) around FMP [16] and is often associated with inflammation of adipose tissue [17], we also examined the impact of baseline body mass index (BMI) on changes in WBC count around FMP.

## Methods

### Study population

The Kailuan cohort study is a community-based and longitudinal health checkup cohort carried out in the Kailuan community in Tangshan, China. It aimed to investigate the risk factors and interventions for cardiovascular and cerebrovascular diseases and other non-communicable diseases by recruiting individuals (≥ 18 years) employed or retired from the Kailuan Group. The cohort design and procedures have been described comprehensively elsewhere [18]. In 2006–2007, 20,400 women were enrolled in 11 hospitals in the Kailuan community for a baseline health checkup (the first checkup). Subsequent checkups were carried up every two years, which involved the administration of questionnaires, clinical examinations, and laboratory tests. Information on menopause was collected through a questionnaire starting from the third checkup, except the fifth checkup. The menopause status of women at the first and second checkups can be inferred from the reported FMP age obtained during the third and subsequent checkups. The final checkup included in this analysis (the seventh checkup) occurred during 2018–2020. Therefore, 16,944 women who provided menopausal information at least once were eligible to participate in the Kailuan ovarian aging study. After excluding those who had not reached their FMP (*n* = 3751), were lost before reaching FMP (*n* = 2320), had no available information on FMP age (*n* = 2706), or had reported hysterectomy or oophorectomy (*n* = 382), 7785 women with FMP age remained eligible for this study.

**Fig. 1:**
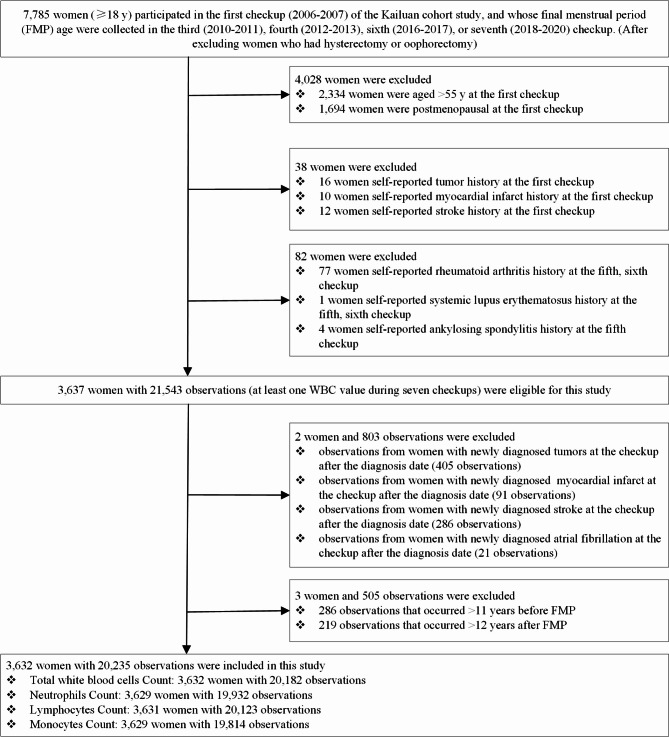
Flowchart of the study population selection. Abbreviations: FMP, final menstrual period; WBC, white blood cells; TWBC, total white blood cells; NEUT, neutrophils; LYM, lymphocytes; MON, monocytes

Next, women who met the following criteria were further excluded: (1) aged > 55 years or were post-menopausal at the first checkup to minimize recall bias; (2) self-reported history of tumors, myocardial infarction, or stroke during the first checkup; (3) self-reported history of rheumatoid arthritis, systemic lupus erythematosus, or ankylosing spondylitis during the fifth and sixth checkup (information on the history of these diseases was only collected during these two checkups). Furthermore, observations from women with newly diagnosed tumors and cardiovascular and cerebrovascular diseases were excluded at the checkup after the diagnosis date. The information on disease diagnosis was obtained from the Municipal Social Insurance Institution and the Hospital Discharge Register, and potential disease information was supplemented via a questionnaire during each checkup [18]. Observations that occurred > 11 years before or > 12 years after FMP (the lower and upper 1% of the time before and after the FMP) were excluded. Ultimately, 3632 women with at least one WBC value, with 20,235 observations, were included. Detailed information on the study sample selection is described in Fig. 1. The number of women eligible for or included in this study at each checkup is shown in Table S1.

This study protocol conforms to the provisions of the Declaration of Helsinki. This study was reviewed and approved by the Ethics Committee of Kailuan General Hospital (protocol number: 2021012). Written informed consent was obtained from the participants.

### Measurement of WBC indicators

We evaluated four peripheral WBC count indicators, including total WBC (TWBC), NEUT, LYM, and MON. Eosinophils and basophils were excluded from the analysis due to their high rate of missing data (> 60%) in the database. At each checkup, venous blood samples were collected in the morning after ≥ 8 h of fasting and transfused into vacuum tube containing EDTA-K2. The blood samples were then subjected to routine blood counts using the Sysmex XT-1800i analyzer and its corresponding reagents (Sysmex, Kobe, Japan) within 2 h of collection at the clinical laboratory of each hospital. The instruments used in all hospitals were of the same model, calibrated uniformly, reagents purchased uniformly, and medical staff trained uniformly.

### Assessment of FMP time

Menopause status and FMP age were self-reported via standardized questionnaires. FMP age was the age at natural menopause (defined as no bleeding for at least one year not attributable to hysterectomy, oophorectomy, or pregnancy) collected at the first subsequent checkup for post-menopausal women. FMP time was the years before or after the FMP age, computed as (age at each checkup – FMP age). Negative and positive values indicated the years before and after FMP age.

### Other variables

Birth date, education level, smoking status (never, previous, or current), alcohol consumption (no, occasional, or often), physical activity (inactive, occasional, active), chronic disease history (hypertension, diabetes, dyslipidemia), and medications were derived from questionnaires administered during the checkups. Height and weight were measured and then calculated as BMI. BMI was categorized into three groups: <24.0 kg/m^2^ (normal weight), 24.0–27.9 kg/m^2^ (overweight), ≥ 28.0 kg/m^2^ (obesity). Blood pressure was measured twice while the participants were seated by trained field workers (i.e., physicians and nurses) using a mercury sphygmomanometer. Blood pressure was the average of 2 measures. Laboratory tests, including total cholesterol (TC), triglyceride (TG), high-density lipoprotein cholesterol (HDL-C), low-density lipoprotein cholesterol (LDL-C), and fasting blood glucose (FBG), were measured by an autoanalyzer (Hitachi 747; Hitachi, Tokyo, Japan) at the central laboratory of the Kailuan General Hospital. Hemoglobin (Hb) is measured in routine blood tests.

Hypertension history and anti-hypertensive medication usage were grouped into four groups: no hypertension history, hypertension history with no medication, hypertension history with medication, and hypertension history with medication missing. This classification was based on the criteria of systolic blood pressure ≥ 140 mmHg and/or diastolic blood pressure ≥ 90 mmHg [19], self-reported physician diagnosis, and current treatment with anti-hypertensive medication. Diabetes history and anti-diabetic medication usage were grouped into four groups: no diabetes history, diabetes history with no medication, diabetes history with medication, and diabetes history with medication missing. This classification was based on the criteria of FBG ≥ 7.0 mmol/L [20], self-reported physician diagnosis, and current anti-diabetic treatment. Dyslipidemia history and lipid-lowering medication usage were grouped into four groups: no dyslipidemia history, dyslipidemia history with no medication, dyslipidemia history with medication, and dyslipidemia history with medication missing. This classification was based on the criteria of TC ≥ 6.2mmol/L, TG ≥ 2.3mmol/L, LDL-C ≥ 4.1mmol/L, HDL-C < 1.0mmol/L [21], self-reported physician diagnosis, and current lipid-lowering treatment.

### Statistical analyses

All analyses were conducted using SAS (version 9.4), except for the Locally Estimated Scatterplot Smoothing (LOESS) analysis, which used R (version 4.1.0). The characteristics of women were presented for each checkup as mean ± SD, median (IQR), or n (%). Significance tests were two-sided, with α = 0.05.

We followed the previous approach to analyze the changes in WBC indicators in relation to the period around FMP age [13–15]. Firstly, we used LOESS with a smoothing set at 0.70 to create plots of repeated measurements for each WBC indicator in relation to the number of years before or after FMP. We visually identified some candidate time-knots that marked changes in the slope, as suggested by the LOESS (Fig. S1). Secondly, we employed piece-wise linear mixed-effect models that included random effects for the intercept and time-knot slopes (Table S2). The most appropriate time-knots were determined based on the lowest Akaike Information Criterion of the model. We identified four time-knots that marked changes in the slope for TWBC, NEUT (TWBC: -6, -3, 0, + 2 years relative to FMP; NEUT: -5, -4, + 2, +6 years relative to FMP). For LYM and MON, we identified two time-knots each (LYM: -5, -1 years relative to FMP; MON: -4, + 2 years relative to FMP). Thirdly, we conducted mixed-effect models using the identified time-knots to estimate the slope of each WBC indicator during specific time segments. Only women with at least two measurements for a particular WBC indicator were included in these models. All models were adjusted for age at checkup (continuous), educational level, smoking status, alcohol consumption, physical activity, hypertension history and anti-hypertensive medication, diabetes history and anti-diabetic medication, dyslipidemia history and lipid-lowering medication, BMI (continuous), and Hb (continuous). Education level and smoking status were used as time-invariant covariates, as they rarely changed during each checkup, while other variables were used as time-varying covariates. Furthermore, to eliminate the potential influence of chronic disease history and medication on the results, we conducted sensitivity analyses by excluding observations from individuals with these conditions. In addition, we excluded observations with WBC counts above the normal reference range for another sensitivity analysis, aiming to minimize the impact of infections condition.

Then, we tested for potential interactions between baseline age (< 45 years and ≥ 45 years) as well as baseline BMI (normal weight, overweight, and obesity) and time segments with mixed-effect models for those WBC indicators. To explore the impacts of baseline age and BMI, we conducted subgroup analyses on WBC indicators with statistically significant interactions.

## Results

### General characteristics

This study included 3,632 women, with 20,235 observations. The median number of checkups was 6 (IQR:5–7). 98.46% of women had ≥ 2 checkups. The actual observations represented 79.6% of the total possible observations. The mean baseline age was 45.59 ± 4.51 years, and the median FMP age was 51 (IQR: 50 ∼ 53). The summary of the observation characteristics at each checkup is shown in Table 1. As this study involved a long-term follow-up study with a large cohort, the number of participating women varied at each checkup, and the available observations decreased from 3339 to 2483. Most women were in the education level of junior high school or below, were non-smokers and non-drinkers. More than half occasionally had physical activity. The average BMI was less than 25 kg/m^2^. The average Hb was increased from 132.75 g/L to 136.25 g/L. From the first to the seventh checkup, the proportion of hypertension, diabetes, and dyslipidemia history tended to increase, and so did the medication.

**Table 1:** Characteristics of the study sample at each checkup (*N* = 3632)

Variables	1st Checkup (2006–2007)	2nd Checkup (2008–2009)	3rd Checkup (2010–2011)	4th Checkup (2012–2013)	5th Checkup (2014–2015)	6th Checkup (2016–2017)	7th Checkup (2018–2020)
**N** ^a^	3339	2984	3151	2993	2703	2582	2483
**Age at checkup**^**b**^, **y**,** mean (SD)**	46.16 (4.10)	47.67 (4.58)	49.40 (4.68)	51.60 (4.68)	53.70 (4.75)	56.34 (4.56)	57.78 (4.45)
**Education level**^**b**^, **n (%)**							
Junior high school or below	2310 (69.22)	1983 (66.45)	2103 (66.74)	1994 (66.62)	1804 (66.77)	1790 (69.35)	1650 (66.51)
Senior high school or above	1027 (30.78)	1001 (33.55)	1048 (33.26)	999 (33.38)	898 (33.23)	791 (30.65)	831 (33.49)
Missing	2	0	0	0	1	1	2
**Smoking status**^**b**^, **n (%)**							
Never	3279 (98.20)	2928 (98.12)	3099 (98.35)	2945 (98.40)	2667 (98.67)	2546 (98.61)	2447 (98.55)
Previous	15 (0.45)	15 (0.50)	12 (0.38)	12 (0.40)	9 (0.33)	8 (0.31)	9 (0.36)
Current	45 (1.35)	41 (1.37)	40 (1.27)	36 (1.20)	27 (1.00)	28 (1.08)	27 (1.09)
**Alcohol consumption**^**b**^, **n (%)**							
No	3104 (92.96)	2728 (91.54)	2973 (94.38)	2831 (94.75)	2549 (94.41)	2443 (94.84)	2336 (94.46)
Occasional	224 (6.71)	240 (8.05)	169 (5.37)	143 (4.79)	139 (5.15)	121 (4.70)	129 (5.22)
Often	11 (0.33)	12 (0.40)	8 (0.25)	14 (0.47)	12 (0.44)	12 (0.47)	8 (0.32)
Missing	0	4	1	5	3	6	10
**Physical activity**^**b**^, **n (%)**							
Inactive	182 (5.47)	541 (18.19)	968 (30.78)	961 (32.21)	869 (32.20)	766 (29.83)	791 (32.00)
Occasional	2828 (84.95)	1887 (63.45)	1767 (56.18)	1703 (57.07)	1556 (57.65)	1522 (59.27)	1426 (57.69)
Active	319 (9.58)	546 (18.36)	410 (13.04)	320 (10.72)	274 (10.15)	280 (10.90)	255 (10.32)
Missing	10	10	6	9	4	14	11
**Diabetes history and anti-diabetic medication**^**b**^, **n (%)**							
No diabetes history	3164 (94.82)	2776 (93.06)	2888 (91.65)	2687 (89.93)	2367 (88.22)	2210 (85.76)	2079 (84.44)
Diabetes history with no medication	131 (3.93)	112 (3.75)	168 (5.33)	204 (6.83)	215 (8.01)	268 (10.40)	262 (10.64)
Diabetes history with medication	42 (1.26)	68 (2.28)	86 (2.73)	82 (2.74)	61 (2.27)	97 (3.76)	99 (4.02)
Diabetes history with medication missing	0 (0)	27 (0.91)	9 (0.29)	15 (0.50)	40 (1.49)	2 (0.08)	22 (0.89)
Missing	2	1	0	5	20	5	21
**Hypertension history and anti-hypertensive medication**^**b**^, **n (%)**							
No hypertension history	2662 (79.77)	2201 (73.81)	2189 (69.47)	1951 (65.21)	1645 (61.15)	1453 (56.41)	1319 (53.34)
Hypertension history with no medication	471 (14.11)	519 (17.40)	676 (21.45)	763 (25.50)	770 (28.62)	877 (34.05)	839 (33.93)
Hypertension history with medication	154 (4.61)	204 (6.84)	274 (8.70)	256 (8.56)	189 (7.03)	241 (9.36)	247 (9.99)
Hypertension history with medication missing	50 (1.50)	58 (1.95)	12 (0.38)	22 (0.74)	86 (3.20)	5 (0.19)	68 (2.75)
Missing	2	2	0	1	13	6	10
**Dyslipidemia history and lipid-lowering medication**^**b**^, **n (%)**							
No dyslipidemia history	2500 (74.87)	1981 (66.39)	1925 (61.09)	1633 (54.58)	1331 (49.30)	1148 (44.46)	973 (39.19)
Dyslipidemia history with no medication	699 (20.93)	995 (33.34)	1199 (38.05)	1320 (44.12)	1298 (48.07)	1428 (55.31)	1387 (55.86)
Dyslipidemia history with medication	10 (0.30)	8 (0.27)	27 (0.86)	17 (0.57)	13 (0.48)	6 (0.23)	48 (1.93)
Dyslipidemia history with medication missing	130 (3.89)	0 (0)	0 (0)	22 (0.74)	58 (2.15)	0 (0)	75 (3.02)
Missing	0	0	0	1	3	0	0
**BMI**^**b**^, **kg/m**^**2**^, **mean (SD)**	24.58 (3.39)	24.56 (3.35)	24.82 (3.36)	24.81 (3.30)	24.79 (3.24)	24.79 (3.35)	24.72 (3.42)
**Hb**,** g/L**,** mean (SD)**	132.75 (13.32)	129.46 (12.98)	133.58 (12.59)	134.60 (11.97)	135.87 (11.47)	136.06 (10.19)	136.25 (9.86)

Abbreviations: SD, standard deviation; BMI, body mass index; Hb, hemoglobin

^a^ N is the number of women whose leukocyte values are available at each examination

^b^ Education level and smoking status were time-invariant variables, and the table showed the data at the first examination. Age at checkup, alcohol consumption, physical activity, hypertension history and anti-hypertensive medication, diabetes history and anti-diabetic medication, dyslipidemia history and lipid-lowering medication, BMI, and Hb were time-varying variables, and the table showed the data at each examination

### Trajectories of WBC indicators in relation to FMP

Table 2 and Fig. S2 show the model-predicted annual changes of WBC indicators in relation to the FMP. For TWBC: there was no statistically significant change up to -3 years relative to FMP; thereafter, the maximum annual decrease was observed from − 3 years to FMP (0.086 × 10^9^/L per year, *P* < 0.0001); this decrease continued during 0 ∼ + 2 years; however, it showed an increase during > 2 years after FMP (0.025 × 10^9^/L per year, *P* = 0.0004). NEUT decreased during − 4 to + 2 years relative to FMP (0.058 × 10^9^/L per year, *P* < 0.0001) while remaining stable during other periods. LYM and MON decreased before − 5 years (0.020 × 10^9^/L per year for LYM, *P* < 0.0001, 0.006 × 10^9^/L per year for MON, *P* < 0.0001). Later, LYM continued to decrease during − 5 ∼ -1 years (0.010 × 10^9^/L per year, *P* < 0.0087) and then flattened out after − 1 years, and MON continued to decrease during − 4 ∼ + 2 years (0.005 × 10^9^/L per year, *P* < 0.0001) and then increased > 2 years after FMP (0.003 × 10^9^/L per year, *P* < 0.0001). In summary, all indicators declined before the FMP. In the post-menopausal period, MON exhibited an increasing trend, along with TWBC.

**Table 2:** Estimated annual changes in WBC indicators within each time segment in relation to the FMP^a^

WBC indicators	Crude model			Adjusted model ^b^						
	Obs. ^c^	β (SE)	*P* Value	Obs. ^c^	β (SE)	*P* Value	A vs. B	B vs. C	C vs. D	D vs. E
							*P* Value ^d^	*P* Value ^d^	*P* Value ^d^	*P* Value ^d^
**TWBC**,** ×10**^**9**^**/L**										
**Time segments relative to FMP**										
A. < -6 y relative to FMP	2,573	0.011(0.013)	0.4014	2,567	0.004(0.014)	0.7944	0.2084	0.0069	0.0325	0.0004
B. -6 ∼ -3 y relative to FMP	3,249	-0.013(0.014)	0.3433	3,222	-0.025(0.015)	0.0909				
C. -3 ∼ 0 y relative to FMP	4,100	-0.060(0.012)	< 0.0001	4,065	-0.086(0.013)	< 0.0001				
D. 0 ∼ + 2 y relative to FMP	2,617	-0.010(0.015)	0.4987	2,597	-0.035(0.016)	0.0256				
E. > +2 y relative to FMP	7,586	0.030(0.004)	< 0.0001	7,428	0.025(0.007)	0.0004				
**NEUT**,** ×10**^**9**^**/L**										
**Time segments relative to FMP**										
A. < -5 y relative to FMP	3,543	0.011(0.009)	0.2026	3,526	0.014(0.010)	0.1546	0.3038	0.3102	< 0.0001	0.4063
B. -5 ∼ -4 y relative to FMP	1,061	-0.026(0.032)	0.4148	1,052	-0.024(0.031)	0.4446				
C. -4 ∼ + 2 y relative to FMP	7,821	-0.050(0.005)	< 0.0001	7,753	-0.058(0.006)	< 0.0001				
D. +2 ∼ + 6 y relative to FMP	4,254	0.003(0.006)	0.6794	4,194	0.005(0.008)	0.5375				
E. > +6 y relative to FMP	3,197	0.009(0.008)	0.2286	3,110	0.015(0.009)	0.0828				
**LYM**,** ×10**^**9**^**/L**										
**Time segments relative to FMP**										
A. < -5 y relative to FMP	3,587	-0.019(0.006)	0.0009	3,570	-0.020(0.004)	< 0.0001	0.0563	0.0041		
B. -5 ∼ -1 y relative to FMP	4,902	0.007(0.003)	0.0210	4,865	-0.010(0.004)	0.0087				
C. > -1 y relative to FMP	11,578	0.012(0.001)	< 0.0001	11,387	0.001(0.003)	0.6530				
**MON**,** ×10**^**9**^**/L**										
**Time segments relative to FMP**										
A. < -4 y relative to FMP	4,584	-0.005(0.001)	< 0.0001	4,566	-0.006(0.001)	< 0.0001	0.8535	< 0.0001		
B. -4 ∼ + 2 y relative to FMP	7,805	-0.003(0.001)	< 0.0001	7,736	-0.005(0.001)	< 0.0001				
C. > +2 y relative to FMP	7,367	0.004(0.000)	< 0.0001	7,213	0.003(0.001)	< 0.0001				

Abbreviations: WBC, white blood cells; TWBC, total white blood cells; NEUT, neutrophils; LYM, lymphocytes; MON, monocytes; Obs., observations; FMP, final menstrual period

^a^ Women with at least 2 observations for a given inflammation marker were included for mixed-effect models

^b^ Adjusted for age at checkup (continuous), educational level, smoking status, alcohol consumption, physical activity, hypertension history and anti-hypertensive medication, diabetes history and anti-diabetic medication, dyslipidemia history and lipid-lowering medication, body mass index (continuous), and hemoglobin (continuous) as fixed effects

^c^ Number of observations used to build models

^d^*P* value for pairwise difference in adjacent time segment slopes

After removing observations of individuals with a history of chronic diseases and medication use and those with a history of chronic diseases but unknown medication use, changes in WBC indicators in relation to FMP remained almost consistent. However, when considering only observations of individuals without a history of chronic diseases, the variations in results were evident in LYM, MON, and TWBC. When observations with WBC counts exceeding the normal reference range were removed, the trajectory of WBC indicators showed stability. Detailed results of sensitivity analysis are shown in Table S3.

### Baseline age effect on WBC indicators trajectories in relation to FMP

The baseline age demonstrated significant interactive effects on changes in WBC indicators during specific time segments around FMP, with TWBC being the exception. Antagonistic interactions were observed during − 4 to + 2 years relative to FMP for NEUT. Conversely, synergistic decreases were observed during − 5 to -1 years relative to FMP for LYM and < + 2 years for MON, and synergistic increase was observed during > + 2 years for MON.

**Fig. 2:**
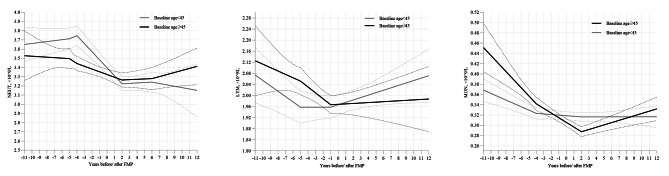
Trajectories of WBC indicators around the final menstrual period in each subgroup of baseline age. Graphs show the estimated means of each WBC indicators by the piece-wise linear mixed-effect model with each model covariate set at its analysis sample mean in each subgroup of baseline age (< 45 years and ≥ 45 years). Models were adjusted for age at checkup (continuous), educational level, smoking status, alcohol consumption, physical activity, hypertension history and anti-hypertensive medication, diabetes history and anti-diabetic medication, dyslipidemia history and lipid-lowering medication, body mass index (continuous), and hemoglobin (continuous) as fixed effects. Solid line and dashed lines represent mean values and 95% CI, respectively. Abbreviations: FMP, final menstrual period; WBC, white blood cells; TWBC, total white blood cells; NEUT, neutrophils; LYM, lymphocytes; MON, monocytes

Further subgroup analysis revealed distinct trajectories of WBC indicator changes around menopause in different baseline age groups (Table 3; Fig. 2). The decrease in NEUT remained consistent during − 4 ∼ + 2 years relative to FMP, but older individuals experienced an increase during > + 6 years. For LYM, younger individuals showed a pattern of decline, stabilization, and subsequent increase. However, older individuals experienced a decline only during − 5 ∼ -1 years relative to FMP, with no significant changes in other time segments. Regarding MON, younger individuals showed a decline during < -4 years relative to FMP, while older individuals experienced an initial decline followed by an increase starting from + 2 years.

**Table 3:** Annual changes in WBC in relation to the FMP across baseline age subgroups ^a^

WBC indicators	Baseline age < 45 years			Baseline age ≥ 45 years		
	Obs. ^d^	β (SE)	*P* Value	Obs. ^d^	β (SE)	*P* Value
**NEUT, ×10** ^**9**^ ** /L**						
**Time segments relative to FMP**						
A. < -5 y relative to FMP	2,918	0.010(0.012)	0.3748	685	-0.005(0.025)	0.8369
B. -5 ∼ -4 y relative to FMP	703	0.033(0.039)	0.3872	356	-0.054(0.057)	0.3471
C. -4 ∼ + 2 y relative to FMP	3,668	-0.087(0.010)	< 0.0001	4,113	-0.030 (0.010)	0.0020
D. +2 ∼ + 6 y relative to FMP	1,562	0.004(0.013)	0.7287	2,588	0.004(0.011)	0.7270
E. > +6 y relative to FMP	538	-0.015(0.025)	0.5424	2,504	0.022(0.010)	0.0338
**LYM**,** ×10**^**9**^**/L**						
**Time segments relative to FMP**						
A. < -5 y relative to FMP	2,957	-0.019(0.005)	0.0003	690	-0.012(0.011)	0.2552
B. -5 ∼ -1 y relative to FMP	2,666	0.000(0.005)	0.9976	2,251	-0.021(0.006)	0.0008
C. > -1 y relative to FMP	3,861	0.009(0.004)	0.0426	7,397	0.002(0.004)	0.7191
**MON**,** ×10**^**9**^**/L**						
**Time segments relative to FMP**						
A. < -4 y relative to FMP	3,614	-0.006(0.001)	< 0.0001	1,036	-0.016(0.004)	< 0.0001
B. -4 ∼ + 2 y relative to FMP	3,658	-0.001(0.001)	0.2648	4,105	-0.009(0.001)	< 0.0001
C. > +2 y relative to FMP	2,068	-0.000(0.001)	0.9775	5,034	0.004(0.001)	< 0.0001

Abbreviations: WBC, white blood cells; NEUT, neutrophils; LYM, lymphocytes; MON, monocytes; Obs., observations; FMP, final menstrual period

^a^ Participants were divided into two baseline age subgroups: Those baseline age < 45 years and those ≥ 45 years. Women with at least 2 measurements for a given lipid parameter were included for mixed-effect models. Models were adjusted for age at checkup (continuous), educational level, smoking status, alcohol consumption, physical activity, hypertension history and anti-hypertensive medication, diabetes history and anti-diabetic medication, dyslipidemia history and lipid-lowering medication, body mass index (continuous), and hemoglobin (continuous) as fixed effects

^b^ Number of observations used to build models

### Baseline BMI effect on WBC indicators trajectories in relation to FMP

Baseline overweight had a synergistic decline in the changes during 0 to + 2 years relative to FMP for TWBC and from − 4 to + 2 years for MON. On the other hand, baseline obesity had a synergistic decline in the changes during − 5 to -1 years relative to FMP for LYM and during < + 2 years for MON.

**Fig. 3:**
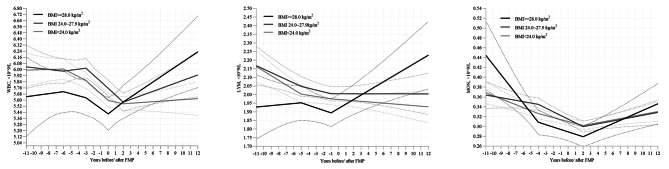
Trajectories of WBC indicators around the final menstrual period in each subgroup of baseline BMI. Graphs show the estimated means of each WBC indicators by the piece-wise linear mixed-effect model with each model covariate set at its analysis sample mean in each subgroup of baseline body mass index (normal weight, overweight, and obesity). Models were adjusted for age at checkup (continuous), educational level, smoking status, alcohol consumption, physical activity, hypertension history and anti-hypertensive medication, diabetes history and anti-diabetic medication, dyslipidemia history and lipid-lowering medication, body mass index (continuous), and hemoglobin (continuous) as fixed effects. Solid line and dashed lines represent mean values and 95% CI, respectively. Abbreviations: BMI, body mass index; FMP, final menstrual period; WBC, white blood cells; TWBC, total white blood cells; NEUT, neutrophils; LYM, lymphocytes; MON, monocytes

The subgroup analysis revealed that different baseline BMI exhibited distinct patterns in WBC indicators changes before and after menopause (Table 4; Fig. 3). In terms of TWBC, individuals with high BMI showed a counteracting effect on the decrease before menopause and demonstrated an increasing trend after menopause. Regarding LYM, normal weight and overweight individuals exhibited consistent changes (decrease during < -5 years relative to FMP), while obese individuals only experienced an increase during > -1 years. As for MON, all individuals in three subgroups experienced an initial decrease followed by an increase. And there were slight variations during <-4 years relative to FMP. Specifically, obese individuals showed a greater decrease during < -4 years, whereas overweight individuals showed no significant change.

**Table 4:** Annual changes in WBC in relation to the FMP across baseline BMI subgroups ^a^

WBC indicators	Normal weight (BMI < 24 kg/m^2^)			Overweight (BMI 24 ∼ 27.9 kg/m^2^)			Obesity (BMI ≥ 28 kg/m^2^)		
	Obs. ^b^	β (SE)	*P* Value	Obs. ^b^	β (SE)	*P* Value	Obs. ^b^	β (SE)	*P* Value
**TWBC, ×10** ^**9**^ ** /L**									
**Time segments relative to FMP**									
A. < -6 y relative to FMP	1,518	0.003(0.018)	0.8689	864	-0.011(0.025)	0.6504	273	0.013(0.046)	0.7747
B. -6 ∼ -3 y relative to FMP	1,658	-0.050(0.019)	0.0094	1,159	0.013(0.026)	0.6192	400	-0.027(0.040)	0.5122
C. -3 ∼ 0 y relative to FMP	2,040	-0.087(0.018)	< 0.0001	1,547	-0.088(0.023)	0.0001	590	-0.070(0.039)	0.0731
D. 0 ∼ + 2 y relative to FMP	1,220	-0.022(0.021)	0.3049	938	-0.091(0.027)	0.0007	356	0.078(0.044)	0.0763
E. > +2 y relative to FMP	3,401	0.007(0.010)	0.4859	2,813	0.036(0.012)	0.0037	1,102	0.066(0.019)	0.0007
**LYM**,** ×10**^**9**^**/L**									
**Time segments relative to FMP**									
A. < -5 y relative to FMP	2,021	-0.027(0.006)	< 0.0001	1,235	-0.020(0.008)	0.0098	391	0.004(0.014)	0.7609
B. -5 ∼ -1 y relative to FMP	2,471	-0.007(0.005)	0.1842	1,789	-0.011(0.006)	0.0976	657	-0.015(0.011)	0.1760
C. > -1 y relative to FMP	5,314	-0.004(0.004)	0.3466	4,276	0.000(0.005)	0.9901	1,668	0.026(0.007)	0.0005
**MON**,** ×10**^**9**^**/L**									
**Time segments relative to FMP**									
A. < -4 y relative to FMP	2,547	-0.006(0.002)	< 0.0001	1,593	-0.003(0.002)	0.1637	510	-0.019(0.005)	0.0003
B. -4 ∼ + 2 y relative to FMP	3,804	-0.004(0.001)	0.0001	2,864	-0.008(0.001)	< 0.0001	1,095	-0.005(0.002)	0.0413
C. > +2 y relative to FMP	3,297	0.003(0.001)	0.0024	2,731	0.003(0.001)	0.0118	1,074	0.007(0.001)	0.0002

Abbreviations: WBC, white blood cells; TWBC, total white blood cells; LYM, lymphocytes; MON, monocytes; Obs., observations; FMP, final menstrual period; BMI, body mass index

^a^ Participants were divided into three baseline BMI subgroups: Normal weight (BMI < 24 kg/m^2^), overweight (BMI 24 ∼ 27.9 kg/m^2^), and obesity (BMI > = 28 kg/m^2^). Women with at least 2 measurements for a given lipid parameter were included for mixed-effect models. Models were adjusted for age at checkup (continuous), educational level, smoking status, alcohol consumption, physical activity, hypertension history and anti-hypertensive medication, diabetes history and anti-diabetic medication, dyslipidemia history and lipid-lowering medication, BMI (continuous), and hemoglobin (continuous) as fixed effects

^b^ Number of observations used to build models

## Discussion

### Main findings

Peripheral WBC is responsible for identifying and eliminating pathogens, clearing damaged cells, and regulating immune responses [22]. They play a crucial role as a vital component of the immune system. This study revealed that WBC counts generally decreased before the FMP, exhibiting diverse patterns of change around FMP. Specifically, LYM experienced a decline during < -1 years relative to FMP; NEUT started to decline approximately 4 years prior to the FMP and remained decreased for up to 2 years post-FMP. A reduction in MON was observed pre-FMP, extending continuously through the two-year period post-FMP. TWBC exhibited a downtrend during − 3 ∼ + 2 years relative to FMP; however, MON and TWBC showed an upward trend during > + 2 years relative to FMP. There was an interaction effect between age and specific time segments around menopause for NEUT, LYM, and MON, and different baseline age subgroups showed distinct trajectories for these three WBC indicators around menopause. Regarding TWBC, LYM, and MON, there was an interaction effect between BMI and specific time segments surrounding menopause. Different baseline BMI subgroups exhibited distinct trajectories for these indicators during the menopausal period.

### Interpretation

Previous studies have shown that changes in reproductive hormones in perimenopausal and post-menopausal women were related to immune aging. Post-menopausal women had higher proinflammatory immune statuses, as demonstrated by altered correlations among NK and T cell subsets, compromised balances between effector T cell subsets, decreased proliferation of T lymphocytes, declined in IFN-c production, declined in NGF expression, and so on [7, 8]. Some studies have analyzed WBC count changes during menopause, but results varied. The analysis of 15,986 women at the American National Health and Nutrition Examination Survey revealed that post-menopausal women had lower numbers of TWBC, NEUT, and MON but higher LYM [9]. A Finnish study divided 1389 women aged 47–55 into pre-menopausal, early-perimenopausal, late-perimenopausal, and post-menopausal groups, finding a decreased number in TWBC and NEUT during menopause, an increase in LYM, and no significant difference in MON [10]. Another study involving 69 women aged 45–60 found that the number of TWBC, LYM, and MON in post-menopausal women was higher than in pre-menopausal women, with no significant difference for NEUT [11]. These studies were cross-sectional comparative studies and did not fully consider the influence of confounding factors such as age and chronic medical history. Our cohort study revealed a decline in WBC counts before FMP, with TWBC, NEUT, and MON remaining decreased for up to 2 years post-FMP. Notably, TWBC and MON exhibited an increase beginning more than 2 years after the FMP. Although there was an increasing trend for LYM beginning from 5 years before FMP in the unadjusted model, this disappeared after multivariate adjustment. In addition, we discovered that chronic diseases influenced the LYM, MON and TWBC trajectories around menopause. Recent studies have reported an increase in TWBC in specific subgroups of individuals with diabetes [23], and dyslipidemia and hypertension were associated with the activation, infiltration, and differentiation of peripheral MON [24, 25]. Furthermore, certain types of LYM levels in the peripheral blood of hypertensive patients were found to be decreased compared to those in healthy individuals [26]. The prevalence of these diseases in women began to increase in middle age [27–29], overlapping with menopause. This may explain the trajectories of LYM, MON, and TWBC observed in this study for individuals with chronic diseases before and after menopause.

The increase in chronological age can lead to immune aging, including changes in the composition, function, and number of immune cells. Animal and human longitudinal studies have indicated that the percentage and number of NEUT tend to increase with age [30, 31]. Our study found that age weakened the decrease in NEUT caused by menopause. Previous studies have shown that the number of peripheral LYM decreased with age [32], and the number of MON was higher in the elderly group compared to the young and middle-aged group [33]. We discovered that age altered the trajectories of LYM and MON around menopause.

The impact of BMI on changes in peripheral immune cells around menopause may be complex. Firstly, with increasing BMI, women’s estrogen levels rise [34, 35], and for a considerable period after menopause, obese individuals have higher estrogen levels than non-obese individuals [16]. This effect may partially counterbalance the decline in estrogen caused by menopause in individuals with a high BMI, as we demonstrated that the impact of menopause on TWBC and LYM was postponed or counterbalanced in overweight or obese individuals compared to those with normal weight. Furthermore, obesity is associated with chronic inflammation. Individuals with high BMI tend to have an increased immune cell [36]. Our findings indicated that overweight or obese individuals experienced an increase in TWBC, LYM, and MON during the post-menopausal stage. However, these increases may be harmful, as obesity affects immune system tissue architecture and leukocyte development, alters the distribution of leukocyte subsets, increases inflammatory phenotypes, and weakens defense against pathogens [37]. Additionally, we discovered a synergistic reduction in the impact of high BMI on the number of immune cells during specific stages of menopause.

### Strengths, significance and limitations

It is the first longitudinal cohort study documenting an association between menopause and peripheral WBC counts, thus providing prospective, population-based cohort evidence. Previous small-scale studies have indicated the association of menopause with diminished immune function and reduced immune cell counts [7, 8, 38]. Nonetheless, this comparative research design fails to adequately distinguish the independent effects of reproductive aging from chronological aging. The advanced immunophenotypic analysis techniques used in these studies, although highly accurate, their prohibitive costs undermine their utility in large-scale, community-based prospective studies. Conversely, some studies find cost-effective blood routine parameters to have high research value. For example, the neutrophil-to-lymphocyte ratio (NLR) may help clinicians diagnose endometriosis and categorize patients into high-risk and low-risk categories, enabling customized follow-up plans [39, 40]; additionally, the NLR serves as an inflammatory biomarker and prognostic indicator for heart failure, cardiovascular disease, and chronic inflammatory diseases [41]. We explored the dynamic changes in immune cell counts around menopause based on blood routine, which is a feasible and economical approach, particularly suitable for implementation in large-scale cohort studies. We used a piece-wise linear mixed-effect model, which had been corroborated by multiple studies [13–15], to distinguish reproductive aging from chronological aging, enhance the validity of our research results, and first depict the trajectories of peripheral WBC counts around menopause. Additionally, this study is the first to report how age and BMI interact with time segments during menopause concerning WBC count changes, revealing that these two factors modify the trajectory of WBC counts at specific time segments of menopause. This finding contributes scientific evidence to support hierarchical immune health management strategies for middle-aged and older Chinese women.

Our findings hold significant implications for women’s health. Firstly, the results contribute to understanding the increased susceptibility to infections in post-menopausal women. Although we observed a slight decline per year in peripheral WBC starting from premenopausal, this decline persists for many years. The menopause period may represent a window of opportunity for implementing interventions to promote immune health. Secondly, our findings underscore the existing recommendation for post-menopausal women to pay attention to their immune health. The US Preventive Services Task Force has issued vaccination recommendations for post-menopausal women [42]. However, our research indicates that the effects of menopause on peripheral WBC counts manifest prior to the FMP. This revelation furnishes a timeline and impetus for immune health management in middle-aged women, advocating for proactive measures (including balanced nutrition, regular exercise, adequate sleep, stress relief, formulation of vaccination plan, etc.) to improve immunity from the early menopause stage. Furthermore, age and BMI should be the key factors for hierarchical management of immune health in middle-aged women.

Our study has several limitations. First, there is the recall bias for FMP age, as it relies on self-reported data obtained through questionnaire surveys, starting from the third checkup onwards. In order to mitigate this bias, elderly and post-menopausal individuals were excluded from the initial checkup. Second, our findings apply only to women experiencing natural menopause. Hormone therapy (HT) is not widely used in China [43, 44], especially among the Kailuan population, where HT is limited to patients undergoing hysterectomy or oophorectomy. These patients were not included in the study. Third, the number of WBC can be affected by many factors, such as lifestyle [45], health conditions and infectious. The Kailuan cohort study required participants to avoid intense exercise and late-night activities in the days preceding their health examination. Blood sampling necessitated a minimum fasting period of 8 h. This study specifically excluded individuals with cardiovascular diseases, tumors, and immune diseases. The analysis further adjusted for lifestyle factors, chronic illnesses, and medication use. Additionally, we excluded observations with WBC counts outside the normal reference range, and the trajectories of WBC indicators showed stability. However, there are still several unexplored factors that may impact the results. Finally, the findings of this single-center population-based study should be generalized cautiously due to its limited representativeness.

## Conclusions

Menopause was associated with changes in peripheral WBC count. The trajectories of various WBC types differ around menopause. There was an interaction effect of age and BMI on the count changes in WBC indicators around menopause. The patterns of change in WBC varied among BMI subgroups and age subgroups before and after FMP. Future research should accumulate long-term cohort data to assess the potential predictive utility of WBC count changes around menopause for infectious diseases, immune related disorders, and the vaccination efficacy.

## Electronic supplementary material

Below is the link to the electronic supplementary material.

Supplementary Material 1

Supplementary Material 2

Supplementary Material 3

## Data Availability

The datasets used and/or analyzed during the current study are available from the corresponding author on reasonable request.

## Abbreviations

BMI: Body mass index
CVD: Cardiovascular disease
E2: Estrogen
MT: Menopause transition
FBG: Fasting blood glucose
FMP: Final menstrual period
FSH: Follicle-stimulating hormone
Hb: Hemoglobin
HT: Hormone therapy
HDL-C: High-density lipoprotein cholesterol
LOESS: Locally Estimated Scatterplot Smoothing
LDL-C: Low-density lipoprotein cholesterol
LYM: Lymphocytes
MON: Monocytes
NEUT: Neutrophils
SWAN: Study of Women’s Health Across the Nation
TC: Total cholesterol
TWBC: Total white blood cells
TG: Triglyceride
WBC: White blood cells
