# Supplementary material for: Trajectories of peripheral white blood cells count around the menopause: a prospective cohort study
Source: BMC Womens Health. 2024 Sep 11;24:504. doi: 10.1186/s12905-024-03344-0 (PMC11389272; doi:10.1186/s12905-024-03344-0)
Supplement: Supplementary file 1 — Supplementary Material 1 [file 12905_2024_3344_MOESM1_ESM.docx]

**Supplemental Figures**

**Fig. S1.** LOESS plots of estimated WBC indicators mean (smoothed at 0.7) in relation to the final menstrual period.

Solid line illustrates mean values. Cross-sectional, 95% CI are indicated by dashed lines.

LOESS, Locally Estimated Scatterplot Smoothing; WBC, white blood cells; TWBC, total white blood cells; NEUT, neutrophils; LYM, lymphocytes; MON, monocytes; FMP, final menstrual period.

**Fig. S2.** Trajectories of WBC indicators around the final menstrual period.

Graphs show the estimated means of each WBC indicators by the piece-wise linear mixed-effect model with each model covariate set at its analysis sample mean. Models were adjusted for age at checkup(continuous), educational level, smoking status, alcohol consumption, physical activity, hypertension history and anti-hypertensive medication, diabetes history and anti-diabetic medication, dyslipidemia history and lipid-lowering medication, body mass index (continuous), and hemoglobin (continuous) as fixed effects. Solid line and dashed lines represent mean values and 95% CI, respectively.

Abbreviations: WBC, white blood cells; TWBC, total white blood cells; NEUT, neutrophils; LYM, lymphocytes; MON, monocytes; FMP, final menstrual period.

**Supplementary Tables**

**Table S1.** Timeline of checkup cycle and the number of women eligible for/ included in this study

| Checkup cycle | Women with FMP age, N (%) | Women included in this study, N (%) | Women with WBC value available, N |
| --- | --- | --- | --- |
| The first Checkup (2006-2007) | 7785 (100) | 3632 (100) | 3339 |
| The second Checkup (2008-2009) | 6654 (85.47) | 3064 (84.36) | 2984 |
| The third Checkup ^a^ (2010-2011) | 6909 (88.75) | 3241 (89.23) | 3151 |
| The fourth Checkup ^a^ (2012-2013) | 6661 (85.56) | 3130 (86.18) | 2993 |
| The fifth Checkup ^a^ (2014-2015) | 5991 (76.96) | 2847 (78.39) | 2703 |
| The sixth Checkup (2016-2017) | 5867 (75.36) | 2810 (77.37) | 2582 |
| The seventh Checkup ^a, b^ (2018-2020) | 6136 (78.82) | 2986 (82.21) | 2483 |

^a^ The menopause information was collected during these checkups;

^b^ The seventh checkup was extended to June 2020 due to the COVID-19 event.

FMP, final menstrual period; WBC, white blood cells.

**Table S2.** AICs of candidate time knots from piece-wise mixed-effect models with random intercept and time knots slopes

| **Knots** | ***AIC*** | ***P* value (null model likelihood ration test)** |
| --- | --- | --- |
| TWBC |  |  |
| Knots = -6, -3, +2 | 64045.7 | <.0001 |
| Knots = -7, -3, +2 | 64052.8 | <.0001 |
| Knots = -5, -3, +2 | 64057.5 | <.0001 |
| **Knots = -6, -3, 0, +2** | **64035.8** | **<.0001** |
| Knots = -5, -3, 0, +2 | 64046.1 | <.0001 |
| Knots = -6, +2 | 64126.9 | <.0001 |
| NEUT |  |  |
| Knots = -6, +2, +6 | 55201.3 | <.0001 |
| Knots = -6, +2, +7 | 55199.6 | <.0001 |
| Knots = -5, +2, +7 | 55188.5 | <.0001 |
| Knots = -5, +2, +6 | 55187.1 | <.0001 |
| Knots = -5, -4, +2, +7 | 55119.7 | <.0001 |
| Knots = -6, -4, +2, +6 | 55120.4 | <.0001 |
| Knots = -6, -4, +2, +7 | 55119.2 | <.0001 |
| **Knots = -5, -4, +2, +6** | **55117.5** | **<.0001** |
| Knots = -5, -3, +2, +6 | 55122 | <.0001 |
| LYM |  |  |
| Knots = -6 | 27587.4 | <.0001 |
| Knots = -5 | 27577.3 | <.0001 |
| Knots = -6, 0 | 27581.2 | <.0001 |
| Knots = -5, 0 | 27578.7 | <.0001 |
| **Knots = -5, -1** | **27575.2** | **<.0001** |
| Knots = -5, +1 | 27580.4 | <.0001 |
| MON |  |  |
| Knots = +2 | -20989.2 | <.0001 |
| **Knots = -4, +2** | **-21085.2** | **<.0001** |

AIC, Akaike Information Criterion; WBC, white blood cells; TWBC, total white blood cells; NEUT, neutrophils; LYM, lymphocytes; MON, monocytes.

**Table S3.** Sensitivity analysis on estimated annual changes in WBC indicators within each time segment in relation to the final menstrual period

| WBC indicators | Sensitivity analysis 1 ^a^ | | |  | Sensitivity analysis 2 ^b^ | | |  | Sensitivity analysis 3 ^c^ | | |
| --- | --- | --- | --- | --- | --- | --- | --- | --- | --- | --- | --- |
|  | Obs. | *β (SE)* | *P* Value |  | Obs. | *β (SE)* | *P* Value |  | Obs. | *β (SE)* | *P* Value |
| **TWBC, ×10^9^ /L** | | | |  |  |  |  |  |  |  |  |
| Time segments relative to FMP | | | |  |  |  |  |  |  |  |  |
| A. < -6 y relative to FMP | 2,505 | -0.002(0.014) | 0.8612 |  | 1,730 | 0.007(0.017) | 0.6895 |  | 2,542 | 0.000(0.013) | 0.9858 |
| B. -6 ~ -3 y relative to FMP | 2,938 | -0.022(0.015) | 0.1487 |  | 1,761 | -0.024(0.019) | 0.2045 |  | 3,125 | -0.028(0.013) | 0.0280 |
| C. -3 ~ 0 y relative to FMP | 3,704 | -0.086 (0.014) | <.0001 |  | 1,891 | -0.072(0.019) | 0.0002 |  | 4,059 | -0.065(0.012) | <.0001 |
| D. 0 ~ +2 y relative to FMP | 2,174 | -0.036(0.017) | 0.0341 |  | 899 | -0.052(0.026) | 0.0441 |  | 2,454 | -0.039(0.015) | 0.0078 |
| E. > +2 y relative to FMP | 6,115 | 0.017(0.007) | 0.0239 |  | 1,878 | 0.002(0.012) | 0.8445 |  | 7,022 | 0.021(0.007) | 0.0013 |
| **NEUT, ×10^9^ /L** | | | |  |  |  |  |  |  |  |  |
| Time segments relative to FMP | | | |  |  |  |  |  |  |  |  |
| A. < -5 y relative to FMP | 3,384 | 0.013(0.010) | 0.2013 |  | 2,266 | 0.020(0.012) | 0.0844 |  | 3,506 | 0.008(0.009) | 0.3756 |
| B. -5 ~ -4 y relative to FMP | 968 | -0.025(0.032) | 0.4354 |  | 596 | -0.019(0.042) | 0.6448 |  | 1,034 | -0.030(0.028) | 0.2758 |
| C. -4 ~ +2 y relative to FMP | 6,856 | -0.059(0.007) | <.0001 |  | 3,346 | -0.064(0.010) | <.0001 |  | 7,640 | -0.053(0.006) | <.0001 |
| D. +2 ~ +6 y relative to FMP | 3,506 | 0.002(0.008) | 0.7769 |  | 1,206 | -0.007(0.014) | 0.6317 |  | 4,053 | -0.004(0.007) | 0.6175 |
| E. > +6 y relative to FMP | 2,490 | 0.004(0.009) | 0.6946 |  | 648 | -0.015(0.016) | 0.2865 |  | 2,969 | 0.012(0.008) | 0.1210 |
| **LYM, ×10^9^ /L** | | | |  |  |  |  |  |  |  |  |
| Time segments relative to FMP | | | |  |  |  |  |  |  |  |  |
| A. < -5 y relative to FMP | 3,424 | -0.021(0.004) | <.0001 |  | 2,303 | -0.022(0.005) | <.0001 |  | 3,506 | -0.017(0.004) | <.0001 |
| B. -5 ~ -1 y relative to FMP | 4,419 | -0.008(0.004) | 0.0335 |  | 2,448 | 0.004(0.005) | 0.4736 |  | 4,768 | -0.007(0.004) | 0.0354 |
| C. > -1 y relative to FMP | 9,541 | 0.001(0.003) | 0.7520 |  | 3,391 | 0.000(0.004) | 0.9948 |  | 10,928 | 0.003(0.003) | 0.3186 |
| **MON, ×10^9^ /L** | | | |  |  |  |  |  |  |  |  |
| Time segments relative to FMP | | | |  |  |  |  |  |  |  |  |
| A. < -4 y relative to FMP | 4,341 | -0.006(0.001) | <.0001 |  | 2,851 | -0.005(0.002) | 0.0008 |  | 4,540 | -0.004(0.001) | <.0001 |
| B. -4 ~ +2 y relative to FMP | 6,846 | -0.005(0.001) | <.0001 |  | 3,340 | -0.003(0.001) | 0.0134 |  | 7,640 | -0.003(0.001) | <.0001 |
| C. > +2 y relative to FMP | 5947 | 0.002(0.001) | 0.0028 |  | 1,831 | 0.000(0.001) | 0.8371 |  | 7,022 | 0.004(0.001) | <.0001 |

^a^ Sensitivity analysis 1 was conducted by excluding observations who had a history of chronic diseases (hypertension, diabetes, and dyslipidemia) with medication and observations who had a history of chronic diseases with medication missing. Models were adjusted for age at checkup(continuous), educational level, smoking status, alcohol consumption, physical activity, hypertension history, diabetes history, dyslipidemia history, body mass index (continuous), and hemoglobin (continuous) as fixed effects.

^b^ Sensitivity analysis 2 was conducted in observations with no history of chronic diseases (hypertension, diabetes, and dyslipidemia). Models were adjusted for age at checkup(continuous), educational level, smoking status, alcohol consumption, physical activity, body mass index (continuous), and hemoglobin (continuous) as fixed effects.

^c^ Sensitivity analysis 3 was conducted by excluding observations where any of the counts for TWBC, NEUT, LYM, or MON exceed the upper limit of the reference range. Models were adjusted for age at checkup(continuous), educational level, smoking status, alcohol consumption, physical activity, body mass index (continuous), and hemoglobin (continuous) as fixed effects.

WBC, white blood cells; TWBC, total white blood cells; NEUT, neutrophils; LYM, lymphocytes; MON, monocytes; FMP, final menstrual period; Obs., observations.
