# Supplementary figures and images for: Trajectories of peripheral white blood cells count around the menopause: a prospective cohort study
Source: BMC Womens Health. 2024 Sep 11;24:504. doi: 10.1186/s12905-024-03344-0 (PMC11389272; doi:10.1186/s12905-024-03344-0)

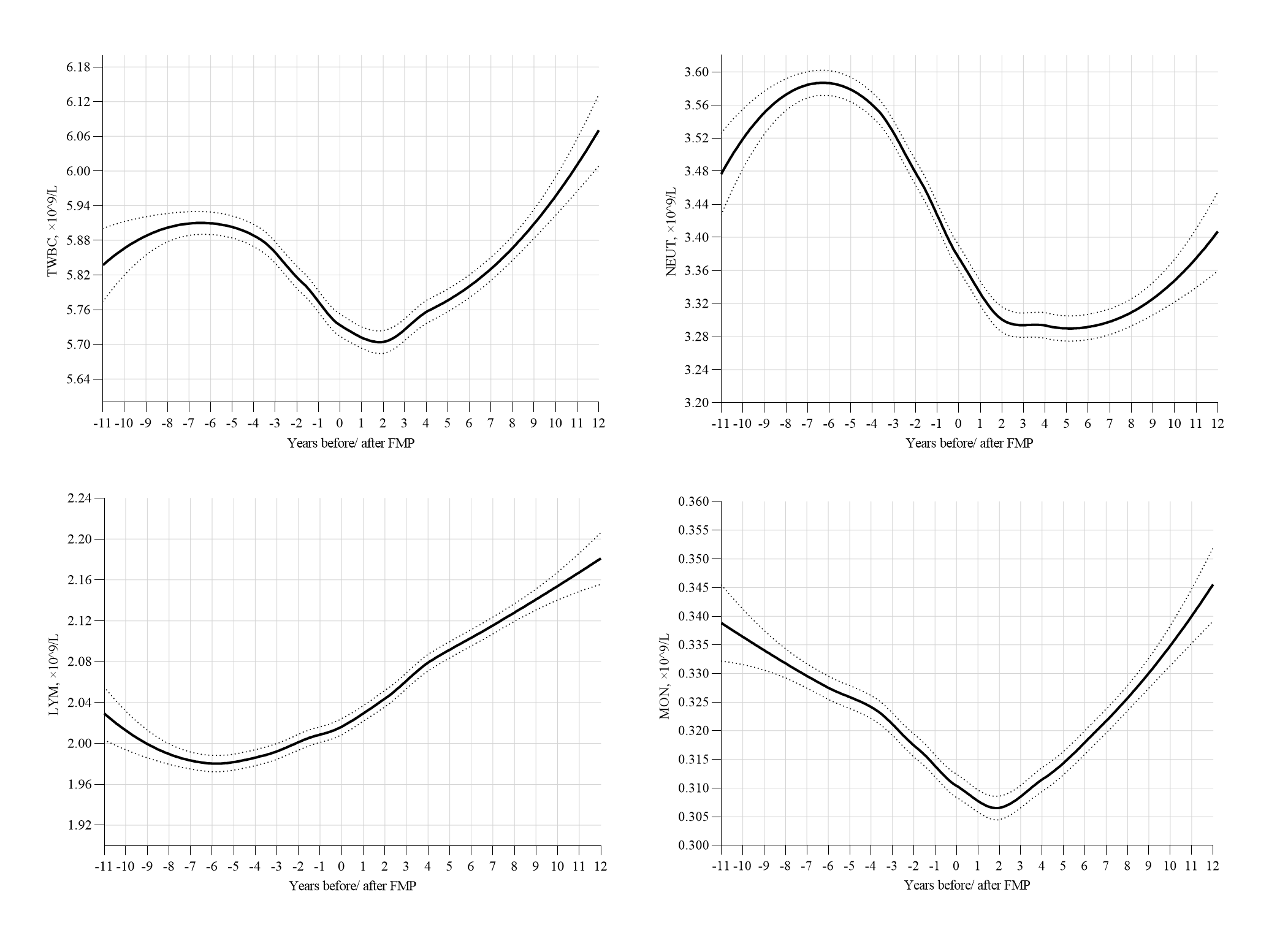

Supplement: Supplementary file 2 — Supplementary Material 2 [file 12905_2024_3344_MOESM2_ESM.tif]

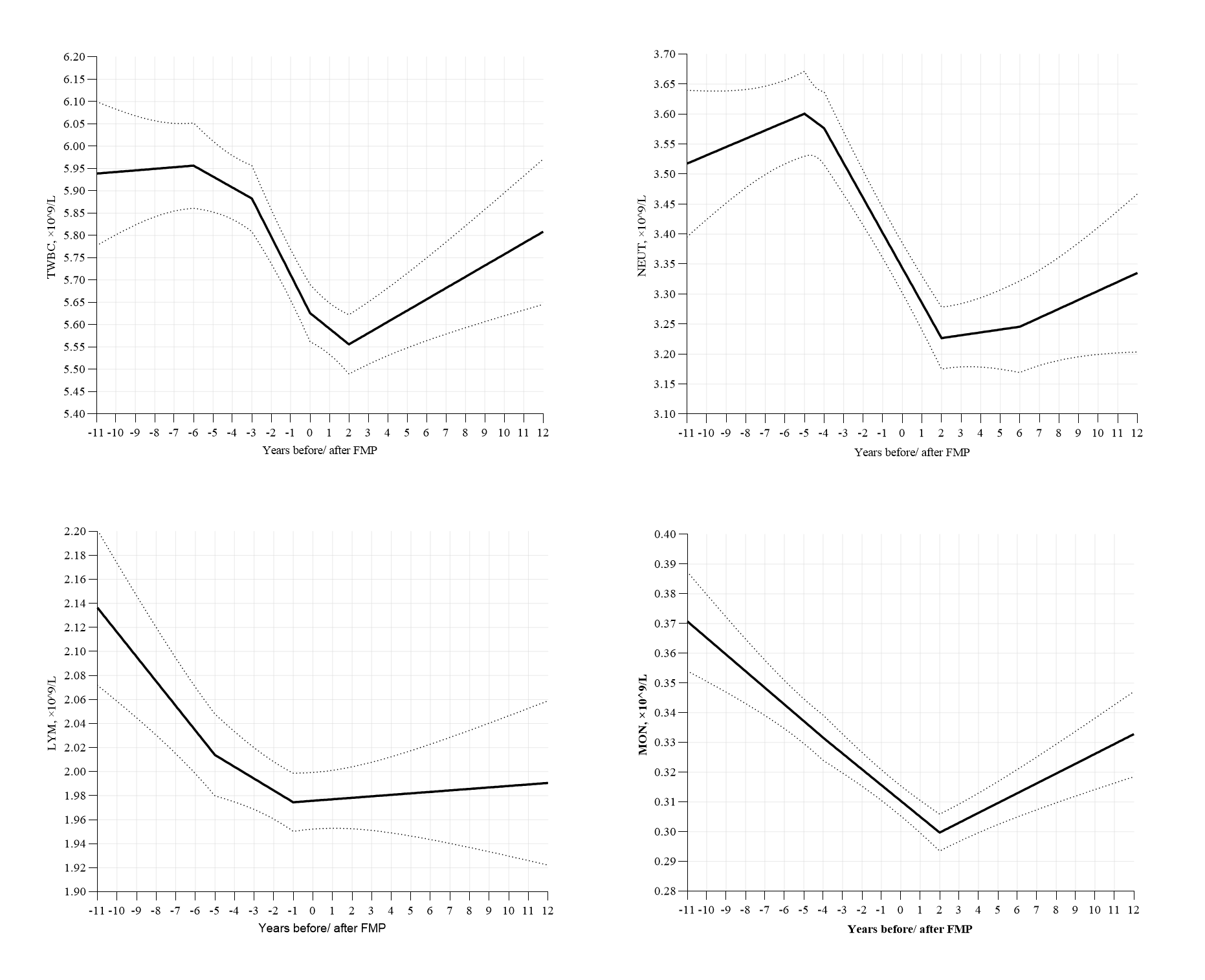

Supplement: Supplementary file 3 — Supplementary Material 3 [file 12905_2024_3344_MOESM3_ESM.tif]
